# A First-Principle Theoretical Study of Mechanical and Electronic Properties in Graphene Single-Walled Carbon Nanotube Junctions

**DOI:** 10.3390/ma10111300

**Published:** 2017-11-13

**Authors:** Ning Yang, Daoguo Yang, Liangbiao Chen, Dongjing Liu, Miao Cai, Xuejun Fan

**Affiliations:** 1 The Department of Mechanical and Electrical Engineering, Guilin University of Electronic Technology, Guilin 541004, China; ldj168168@126.com (D.L.); caimiao105@gmail.com (M.C.); 2 The Department of Mechanical Engineering, Lamar University, Beaumont, TX 77706, USA; lchen3@lamar.edu (L.C.); xfan@lamar.edu (X.F.)

**Keywords:** junctions, graphene-carbon nanotubes, mechanical strength, electric performance, first-principles theory

## Abstract

The new three-dimensional structure that the graphene connected with SWCNTs (G-CNTs, Graphene Single-Walled Carbon Nanotubes) can solve graphene and CNTs′ problems. A comprehensive study of the mechanical and electrical performance of the junctions was performed by first-principles theory. There were eight types of junctions that were constituted by armchair and zigzag graphene and (3,3), (4,0), (4,4), and (6,0) CNTs. First, the junction strength was investigated. Generally, the binding energy of armchair G-CNTs was stronger than that of zigzag G-CNTs, and it was the biggest in the armchair G-CNTs (6,0). Likewise, the electrical performance of armchair G-CNTs was better than that of zigzag G-CNTs. Charge density distribution of G-CNTs (6,0) was the most homogeneous. Next, the impact factors of the electronic properties of armchair G-CNTs were investigated. We suggest that the band gap is increased with the length of CNTs, and its value should be dependent on the combined effect of both the graphene’s width and the CNTs’ length. Last, the relationship between voltage and current (U/I) were studied. The U/I curve of armchair G-CNTs (6,0) possessed a good linearity and symmetry. These discoveries will contribute to the design and production of G-CNT-based devices.

## 1. Introduction

The novel three-dimensional structure that graphene connected with carbon CNTs (G-CNTs) is currently receiving much attention. Like graphene and CNTs, the G-CNTs has excellent mechanical and electrical properties. Furthermore, it can solve the problems of the agglomeration of graphene and the intertwining of CNTs. Thus, G-CNTs have been used in many applications such as transparent conductors [1], supercapacitors [2], batteries [3], biosensors [4], and gas detection [5]. However, the junctions are very complex and diverse; they can be composed of the graphene with different chirality and CNTs with different diameters. Different connections make the junctions different; however, we still do not know much about their mechanical and electrical properties, such as their mechanical strength, band gap, and current characteristic. Therefore, with the development of G-CNT-based devices, the research on the junctions between graphene and CNTs will receive continuous attention.

At present, graphene and CNTs are the most promising 2-D materials for the industry [6,7]. They have excellent mechanical and electrical properties [8,9]. Moreover, they have a large surface area ratio and excellent optical and heat dissipation performance [10,11,12]. However, the development of graphene and carbon nanotubes is also faced with many problems. We know that graphene is a completely 2D material but it is very easy to be agglomerated in its natural state owing to its sheet structure [13]. In this case, the performance of graphene is seriously affected. For example, the agglomeration causes a great reduction in graphene’s surface area, which has a negative impact on sensitive materials; this is bad for the application and development of the sensor [14]. As for CNTs, they are easily intertwined [15]. Obviously, this can change the electric properties of CNTs, which is unfavorable for the integrated circuit, electrode, and transistor [16]. In such circumstances, a candidate material which can solve the above problems is needed urgently. Thus, the 3D interconnected material of G-CNTs is produced, which can solve the problems of agglomeration and intertwine, and retain good performance [17]. Additionally, the G-CNTs can also increase the surface area ratio [18]. These properties are very beneficial for gas adsorption [19] and energy storage [20]. Some research has shown the G-CNTs were used in capacitors owing to their large capacitance. Gupta et al. succeeded in fabricating the hybrid multilayers of graphene/multi-walled carbon nanotubes via electrostatic layer-by-layer self-assembly for high-performance integrated electrochemical energy storage [21]. Beidagh et al. found that the micro-supercapacitor based on interdigitated electrodes of G-CNTs can possess ultra-high-power handling performance [22]. Wang et al. used a new material architecture of ruthenium oxide anchored graphene and CNT hybrid foam to develop a supercapacitor that showed superior gravimetric and per-area capacitive performance [23]. In addition, the capacitors based on graphene/MnO_2_/CNTs nanocomposites [24,25,26] were also reported. On the other hand, this interconnected material also has wide application prospects in the battery, transparent conductor, gene transfection, and bifunctional catalysts. Ryoo et al. found that G-CNTs held high biocompatibility for NIH-3T3 fibroblasts showing high potential for bioapplications, such as proliferation, focal adhesion, and gene transfection [27]. Zhao et al. fabricated the G-CNTs by a facile catalytic growth on layered double hydroxide that could be used in high-rate Li-S Batteries [28]. Tung et al. used G-CNTs to design high-performance transparent conductors in low temperature whose transmittance can be up to 85% [1]. 

Although G-CNTs have a bright future in many areas, research on them is still relatively scarce, which leads to the lack of adequate theoretical guidance in many applications. With the change of the chirality and diameter, the properties of CNTs are different. Furthermore, the properties of graphene change with chirality and side length. However, the impacts of these factors on G-CNTs’ performance are still not clear. Although some studies have reported structures and properties of the G-CNTs, the reports lack detail. There are fewer studies on the comprehensive study of the connection ways, mechanical strength, and electric properties. The capacitor, battery, gas adsorption, and many other aspects of G-CNTs were analyzed; however, the studies were experimental and macroscopical. Therefore, the study on the connected ways responding to mechanical and electrical properties on a Nano level is essential and significant. In this article, we used the two methods of density functional theory (DFT) [29] and density functional based tight binding (DFTB) [30,31] to study intrinsic characteristics of G-CNTs. It is known that DFT has been previously applied in the calculation of various kinds of systems. However, nanostructures and biomolecules are often too large for DFT, but their electronic properties are still of interest; hence, the quantum mechanical description is needed. In addition, the classical force field can handle large systems but is missing quantum mechanics. Nevertheless, empirical tight binding can not only describe the quantum mechanics but also has been applied to systems up to a few million atoms. Therefore, because of superiority, DFTB has been successfully applied in various fields. For example, DFTB has been used successfully to model the electron transport in molecular nanostructures [32]. DFTB treatment of dispersive Van der Waals interactions in DNA modeling has been presented by Elstner [33]. In addition, a spin-polarized extension of DFTB has been described in detail in a study of iron clusters [34]. Moreover, DFTB was successfully used in the calculations of graphene [35], CaCO_3_ [36], SOI MOSFETs [37], and many other applications. Therefore, the methods of DFT and DFTB were used in our study for the calculations of different G-CNTs.

In this paper, the different connected ways were discussed via DFT, such as the structures of armchair and zigzag graphene connected (3,3), (4,0), (4,4), and (6,0) CNTs (arm-33, arm-40, arm-44, and arm-60; zig-33, zig-40, zig-44, and zig-60), respectively. We found that the structure of arm-60 was the best also had the best electrical performance. Then, we used DFTB to investigate the relationship of band gap with the length of CNTs and the side length of graphene and the curve of voltage and current (U/I). The same result occurred; the current characteristic of arm-60 was the best. On the other hand, the band conductance is nearly independent of the tube length but changes strongly with the link structures.

## 2. Results and Discussion

### 2.1. The Properties and Stability of the Eight Types of G-CNTs

**Figure 1:**
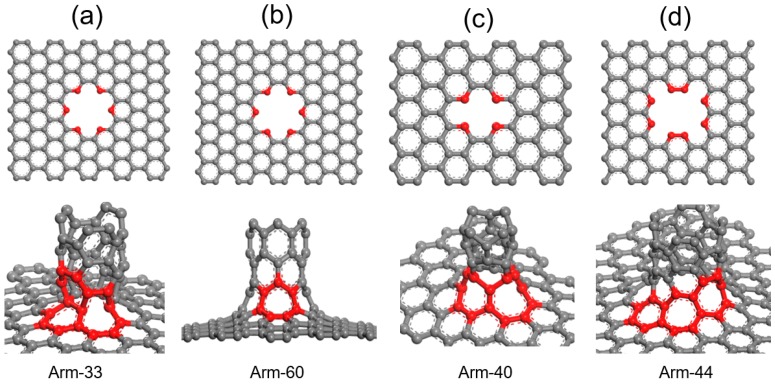
The interconnected graphene and optimized structures of graphene single-walled carbon nanotubes (G-CNTs) of arm-33 (**a**), arm-60 (**b**), arm-40 (**c**), and arm-44 (**d**), respectively. The atoms marked red in graphene are ready to connect with carbon nanotubes (CNTs) to form a carbon ring, the red carbon ring is the linkage of graphene and CNTs.

There are many interconnected ways between graphene and CNTs. Different ways may have different performances. Generally, the pristine (3,3), (4,0), and (4,4) CNTs and the zigzag graphene with the short side length is semimetal while (6,0) CNTs and armchair graphene show metallicity. Hence, owing to their different electrical performances, the (3,3), (4,0), (4,4), and (6,0) CNTs and the zigzag and armchair graphenes were chosen to interconnect respectively, and then to discuss their structures and properties. The structures of the zigzag and armchair graphenes are the same, but the crystal orientation is different, thus taking armchair graphene as an example. The Figure 1 shows the interconnected structure of arm-33, arm-40, arm-44, and arm-60, respectively. The upper part of Figure 1 represents the link structure of graphene, in which the atoms marked red are ready to connect with CNTs to form a carbon ring. There are six interconnected atoms in arm-33 and arm-60 while there are four and eight interconnected atoms in arm-40 and arm-44, respectively. In fact, the number of the interconnected atoms in graphene depends on the shape of the cross section in CNTs. The underpart of Figure 1 shows the optimized structure G-CNTs, in which the red atoms represent the carbon rings that link the graphene and CNTs. Obviously, the interconnected part of the graphene is raised. Arm-33 will form two kinds of carbon rings; one is a hexagon, and the other is an octagon. However, the structure of the octagon ring is irregular. There is only one carbon ring in arm-60, and the heptagon carbon ring is very smooth. This is similar to the research of Baowan; the structure of arm-60 is the most symmetrical, in which the carbon ring is just like that of graphene [38]. Arm-40 has two kinds of carbon rings that have seven and eight atoms, respectively. As for arm-44, there are three kinds of carbon rings that have six, seven, and seven atoms, respectively. It should be noted that the position of the two heptagon rings is different. In comparison, the structure of arm-60 is the smoothest and its deformation is the smallest.

In order to further understand the stability of these interconnected structures, the binding energy was calculated here. Binding energy is a good way to reflect the bonding strength of atoms. The bigger the binding energy is, the better the bonding strength of atoms and the stability of structure will be. Therefore, the stability of the target structure can be analyzed by the binding energy. Here, we calculated the average binding energy of the interconnected part. The binding energy (*E*_b_) can be defined by Equation (1).

*E*_b_ = [*E*_graphene+CNT_ − (*E*_graphene_ + *E*_CNT_)]/*n* (1)

where the *E*_graphene+CNT_ is the total energy of graphene-CNT structure, the *E*_graphene_ is the energy of connected graphene, and the *E*_CNT_ is the energy of the connected CNTs. The *n* is the number of connected atoms. The bonding energy, band gap, bond length, and the height of the bulge of graphene in the armchair and zigzag G-CNTs (A-G-CNTs and Z-G-CNTs) were listed in Table 1.

From the analysis of Table 1, the binding energy of arm-60 is the biggest: its value is 4.06 eV, while the minimum energy is 3.26 eV, which occurred in a zig-40. In addition, compared with A-G-CNTs and Z-G-CNTs, the binding energy A-G-CNTs is larger than that of Z-G-CNTs. Therefore, the interconnected structure of A-G-CNTs is more stable. However, we should note that all the binding energy is acceptable [39,40,41]. Shahsavari found that by introducing heptagonal and octagonal rings in the junctions, the G-CNTs exhibited strong out-of-plane properties [17]. This agrees with the structures we analyzed. The band gap is smaller than that of the zigzag and is the smallest in arm-60 with 0 eV. We believe that this is because the interconnected structures of A-G-CNTs are more stable, and the structure of arm-60 is an ideal mode. These results explained by the band gap agrees with the binding energy. Generally, the bond length of C-C is 1.42 Å [42]. The bond length of arm-60 and zig-60 are 1.421 and 1.419 Å, respectively. They are very close to the normal value of graphene and CNTs. The bond length of the rest of the G-CNTs is longer than the normal value, but they still belong to the normal range [43]. By comparing the A-G-CNTs with the Z-G-CNTs, the height of the bulge of graphene in A-G-CNTs is generally taller. Here, it should be noted that the height of arm-60 and arm-44 is relatively taller, and the values are 2.382 and 2.464 Å, respectively. In fact, the height is closely dependent on the linkage part of G-CNTs. We suggest that this is because the diameters of (6,0) and (4,4) CNTs are close to the linkage part of graphene, hence the interconnected structures of arm-60 and arm-44 are smoother. Furthermore, their bulge occurs in the whole graphene and is homogeneous. However, the diameter of the (4,4) CNTs is bigger than that of (6,0) CNTs, and there are three linked carbon rings in arm-44. Thus, the height of the bulge of graphene in arm-44 is the tallest, while the interconnected structures of arm-60 is still smoothest. As for arm-33 and arm-40, the bulge just occurs in a partial part due to the bad linkage, hence the height is smaller.

### 2.2. The Impact Factors on the Electronic Properties of G-CNTs

**Figure 2:**
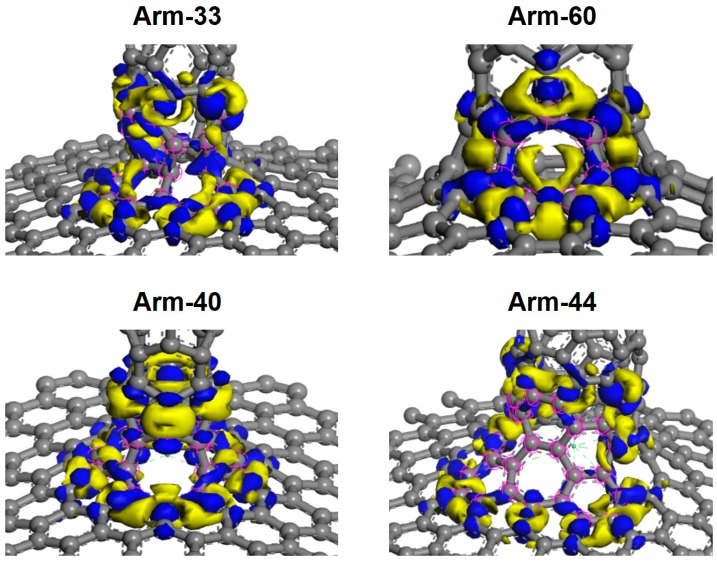
The charge of density difference of arm-33, arm-60, arm-40, and arm-44 respectively.

In this section, the charge density difference was discussed to further study the case of interconnected structures. The electron transfer was used to analyze the quality of their links. Because of the superiority of A-G-CNTs, it was taken as an example. Figure 2 shows the charge of density difference of arm-33, arm-60, arm-40, and arm-44, respectively. It is obvious that the charge density distribution of the interconnected part of arm-60 is the most homogeneous, and the electron transfer is similar to graphene and CNTs. This means that the interconnection structure of arm-60 is stable. The charge density distribution of the interconnected parts of arm-33 and arm-40 is not very good and some atoms lack charge distribution. The charge density distribution of arm-40 is the worst. Based on the above analysis, the distribution of the charge density had demonstrated that interconnection structure of arm-60 was the best.

**Figure 3:**
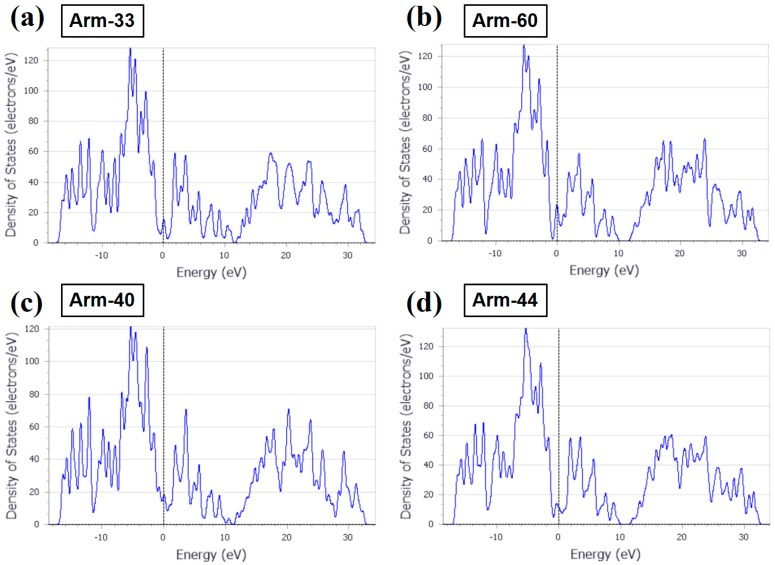
The density of states (DOS) of (**a**) arm-33, (**b**) arm-60, (**c**) arm-40, and (**d**) arm-44, respectively. The dotted line represents the Fermi level.

The density of states (DOS) of arm-33, arm-60, arm-40, and arm-44 are analyzed too, as shown in Figure 3. Basically, the maximum energies of these four models are the same, and the maximum energies are approximately 120 eV. Moreover, the DOS shape in the Fermi level of arm-33 is similar to that of arm-44, whereas the DOS shape in the Fermi level of arm-40 is similar to that of arm-60. The result is caused by the effect of CNTs. The (3,3) and (4,4) CNTs and the (4,0) and (6,0) CNTs are the same type of CNTs, respectively. In addition, it should be noted that the energy in the Fermi level of arm-40 is comparatively high. On the whole, these four models have good electrical properties and show some delocalization. The delocalization in arm-60 is the biggest.

**Figure 4:**
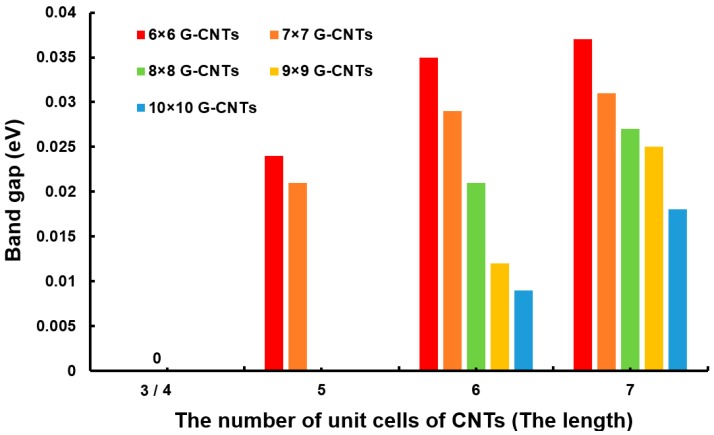
The band gap of different kinds of arm-60. The sizes of the graphene are 6 × 6-, 7 × 7-, 8 × 8-, 9 × 9-, and 10 × 10- unit cells, and the lengths of (6,0) CNTs are 3-, 4-, 5-, 6-, and 7- unit cells, respectively.

Next, the impact factors of the electronic properties of G-CNTs were investigated. It is well known that the electric properties of CNTs depend on diameter and length, and the electric properties of graphene are related to their side length [44,45]. Therefore, the band gap of G-CNTs (different side length of graphene and different length and diameter of CNTs) were calculated in this section. According to the previous analysis, the interconnected structure of arm-60 was an ideal model; thus, it was chosen as the object of analysis. The band gaps of arm-60 with different size are shown in Figure 4. The sizes of the graphene are the supercells of 6 × 6, 7 × 7, 8 × 8, 9 × 9, and 10 × 10, and the lengths of (6,0) CNTs are 3-, 4-, 5-, 6-, and 7-unit cells, respectively. What kind of changes will the length make? First, all the band gaps are zero eV when the CNTs has 3-unit cells. The band gaps of 6 × 6 and 7 × 7 G-CNTs arise a small opening starting from 4-unit cells, whereas 8 × 8, 9 × 9, and 10 × 10 G-CNTs have a small opening starting from 5-unit cells. Moreover, the band gaps of 6 × 6 and 7 × 7 G-CNTs are relatively bigger. Generally, the graphene with the bigger size would have a better electrical performance [46]. We think this character also applies to G-CNTs. In addition, it is obvious that with the increase of the length of the CNTs, the band gaps get bigger and bigger. Novaes et al. found that, for metallic CNTs, the conductance is nearly independent of the tube length, but changes strongly with the link structure, while the opposite occurs for semiconducting CNTs [47]. The (4,0) CNTs is semiconducting. The (3,3), (6,0), and (4,4) CNTs should be metallic, however, owing to their small diameter, they still have quite a small energy gap; as a result, they are semi-metal. Thus, our results still agreed with Novaes’ study. Based on the above, we suggest that the band gap is increased with the length of CNTs, but its value should depend on the combined effect of both the graphene’s width and CNTs’ length.

### 2.3. The Relationship between Voltage and Current of Armchair G-CNTs

**Figure 5:**
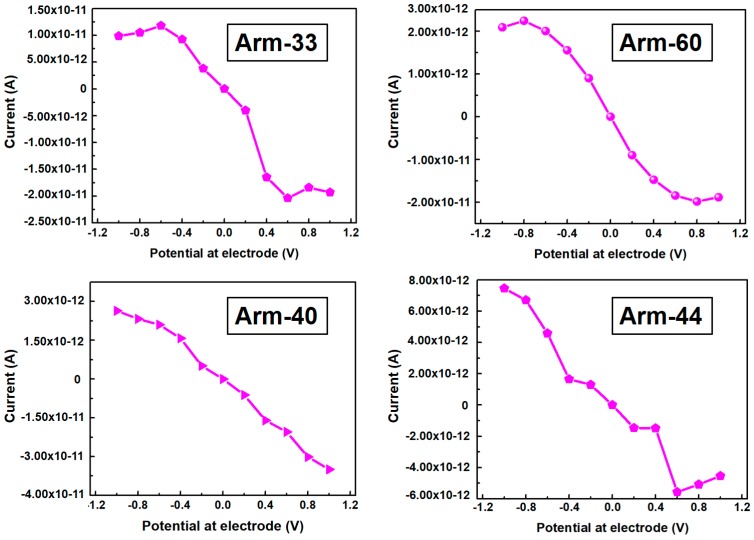
The curve of voltage and current (U/I) of arm-33, arm-60, arm-40, and arm-44 respectively.

Last, the U/I curve of arm-33, arm-60, arm-40, and arm-44 were analyzed by DFTB, as shown in Figure 5. The applied voltage was arranged from −1 to +1 eV. First of all, the U/I curve of arm-60 can be found intuitively that its linearity is good, and it possesses a good symmetry while the linearity of arm-33 and arm-44 is bad. These are agreed with the results of charge density difference we discussed. In addition, arm-60 has the biggest electric current under the same voltage. However, it should be noted that the current of the positive voltage is bigger than that of the negative voltage of other three kinds of interconnection structures. This is because of the instability of the linkage part. Therefore, these results can prove the superiority of arm-60.

## 3. Theory and Simulations

The calculations of the junctions of G-CNTs were performed by DFT, a kind of quantum mechanics researching for the electronic structure of the multi-electron system. It has been widely used in the study of physical and chemical properties, including nanomaterials of graphene and carbon nanotubes [48,49]. DFT can accurately simulate hundreds of atomic systems and describe the atom as quantum particles, namely the set of the nucleus and the electrons [50]. The Time-Independent Schrodinger’s equation is the key of the DFT, which can be described as Equations (2) and (3).

*HΦ*[{*R*_I_, *r*}] = *E*_tot_*Φ*[{*R*_I_, *r*}] (2)

*H* = *P*_I_^2^/2*M*_I_ + *Z*_I_Z_J_ e^2^/***R***_IJ_ + *p*^2^/2*m*_e_ + e^2^/**r** − *Z*_I_e^2^/|***R***_I_ − **r**| (3)

where the ***R*** and ***r*** is the coordinate of atomic nucleus and electrons, respectively. The generalized gradient approximation (GGA) and local density approximation (LDA) are the functionals commonly used in quantum mechanics calculation. Generally, LDA fails to accurately determine a weak van der Waals interaction, which usually significantly overestimates the magnitude of a weak interaction. Consequently, GGA was chosen in this work and it can be described as Equation (4).

*E*_xc_[*ρ*] = ∫*f*_xc_[*ρ*(***r***), |*∆ρ*(***r***)|] d***r*** (4)

where the exchange and correlation energy in inhomogeneous electron gas is replaced by the *E*_xc_[*ρ*] in uniform electron gas.

The calculations of the relationship of band gap with the length of CNTs and the side length of graphene and the curve of U/I were performed by DFTB. In DFTB theory, the total energy of it can be described as Equation (5). After five transformations, the expression can be converted to Equation (6):

(i) Expanding the Kohn-Sham total energy expression of DFT to 2nd order in terms of electron and magnetization density fluctuations;
(ii) Representing the Hamiltonian elements in a minimal basis of pseudo-atomic orbitals;
(iii) Expressing the charge density in terms of Mulliken charges;
(iv) Expand the magnetization density in terms of non-overlapping spherically symmetric functions;
(v) Replacing the remaining terms with a short range repulsive energy. The reader interested in it can read Frauenheim’s study [31].

(5) $$E_{tot}=\sum_{\sigma =↑,↓}.\;\sum_{i}^{occ}n_{i\sigma }\left\{\langle \varphi_{i\sigma }\left|-\frac{\nabla^{2}}{2}+v+\frac{1}{2}\int \frac{n\left(r^{\prime }\right)d^{3}r^{\prime }}{\left|r-r^{\prime }\right|}\right|\varphi_{i\sigma }\rangle \right\}+\sum_{xc}\left[n,\mu \right]+\frac{1}{2}\sum_{\alpha \beta }^{N}\frac{Z_{\alpha }Z_{\beta }}{R_{\alpha }-R_{\beta }}$$

(6) $$E_{tot}=\sum_{\sigma =↑,↓}.\;\sum_{i}^{occ}n_{i\sigma }\left\{\sum_{\mu }.\sum_{v}c_{\mu i\sigma }c_{vi\sigma }H_{\mu v}^{O}\right\}+\frac{1}{2}\sum_{\alpha }.\sum_{\beta }\gamma_{\alpha \beta }\Delta_{q_{\alpha }}\Delta_{q_{\beta }}+\frac{1}{2}\sum_{\alpha }^{N}.\sum_{l\epsilon \alpha }.\sum_{l^{\prime }\epsilon \alpha }p_{\alpha l}p_{\alpha l^{\prime }}W_{\alpha ll^{\prime }}+E_{rep}$$

In this calculation, the ultrasoft pseudo potential was used to describe the interaction between electrons and ions. The cutoff energy was 340 eV, and the Brillouin zone was sampled using a 9 × 9 × 1 Monkhorst-Pack [51] k-point grid and the Methfessel-Paxton [52] smearing was 0.05 eV. The convergence criterion of self-consistent field energy was 1.0 × 10^−6^ eV, and the MAX force was 0.03 eV/Å.

## 4. Conclusions

In this paper, different kinds of G-CNTs were investigated to find a stable structure with excellent performance via DFT and DFTB. Generally, the binding energy of A-G-CNTs was stronger than that of Z-G-CNTs. Moreover, arm-60 had the biggest binding energy, while zig-40 had the smallest one. At the same time, the charge density distribution of arm-60 was the most homogeneous. Compared with the band gap between A-G-CNTs and Z-G-CNTs, it was found that the electrical performance of A-G-CNTs was better than that of Z-G-CNTs. Additionally, the band gap was increased with the length of CNTs, and its value should be dependent on the combined effect of both graphene’s width and CNTs’ length. Finally, the U/I curve of arm-60 had a good linearity, and it possessed good symmetry. In addition, arm-60 has the biggest electric current under the same voltage. These discoveries provide a strong support for better application of G-CNTs, such as sensors, transparent conductors, supercapacitors, and batteries.

## Figures and Tables

**Table 1:** The bonding energy, band gap, bond length, and height of armchair and zigzag G-CNTs.

Intrinsic Characteristics	Armchair G-CNTs					Zigzag G-CNTs		
	33	60	40	44	33	60	40	44
Bonding Energy (eV)	3.71	4.06	3.40	3.63	3.54	3.91	3.26	3.54
Band Gap (eV)	0.139	0	0.011	0.047	0.222	0.021	0.160	0.130
Bond Length (Å)	1.515	1.421	1.474	1.474	1.513	1.419	1.463	1.474
Height (Å)	2.098	2.382	2.141	2.464	1.977	2.263	1.221	1.921
